# Assessment of the correlation between residual hearing and audiologic outcomes after cochlear implantation in patients with cochlear nerve deficiency

**DOI:** 10.1002/lio2.888

**Published:** 2022-08-19

**Authors:** Simeng Lu, Xingmei Wei, Ying Kong, Biao Chen, Jingyuan Chen, Lifang Zhang, Mengge Yang, Sha Liu, Yongxin Li

**Affiliations:** ^1^ Department of Otorhinolaryngology Head and Neck Surgery, Beijing Tongren Hospital Capital Medical University Beijing China; ^2^ Key Laboratory of Otolaryngology Head and Neck Surgery, Ministry of Education, Beijing Institute of Otolaryngology, Beijing Tongren Hospital Capital Medical University Beijing China

**Keywords:** cochlear implant, cochlear nerve deficiency, residual hearing

## Abstract

**Objective:**

This study aimed to assess the correlation between residual hearing and audiologic outcomes after cochlear implant (CI) surgery in patients with cochlear nerve deficiency (CND).

**Methods:**

This retrospective study included 57 patients with CND who underwent CI surgery. Patients were divided into four groups according to hearing level (80–95, 95–110, 110–120, and >120 dB) and three groups according to residual hearing (entire spectrum hearing, partial spectrum hearing, and no spectrum hearing) based on the measured response at each frequency. Auditory performance (categorical auditory performance [CAP], Infant–Toddler Meaningful Auditory Integration Scale [IT‐MAIS]) and speech perception (speech intelligibility rating [SIR] and meaningful use of speech scale [MUSS]) were assessed before and 2 years after the surgery.

**Results:**

Forty‐seven (82.5%) patients had complete or total hearing loss (≥95 dB) and 17 (29.8%) had no spectrum hearing before CI surgery. Twenty‐nine (50.9%) patients did not exhibit residual hearing at 4 kHz. All patients demonstrated an improvement in auditory performance and speech perception: the CAP score in the 80–95 dB group was significantly higher than that in the 110–120 and >120 dB groups, and the entire spectrum hearing group showed significantly higher CAP, SIR, and IT‐MAIS scores than the partial spectrum hearing group and significantly higher CAP, SIR, IT‐MAIS, and MUSS scores than the no spectrum hearing group.

**Conclusion:**

For patients with CND, residual hearing, especially high‐frequency residual hearing, was poor and postoperative audiologic outcomes were significantly associated with the range of residual hearing.

**Level of Evidence:**

4

## 1 INTRODUCTION

The incidence of congenital sensorineural hearing loss (SNHL) is estimated to be 0.1%–0.4%,^1^ while the prevalence of cochlear nerve deficiency (CND) in children with congenital SNHL is estimated to be 18%.^2^ CND is defined as either a thin cochlear nerve (CN) or no CN observed on three‐dimensional (3D) magnetic resonance imaging (MRI). The presence of a thin CN is referred to as cochlear nerve hypoplasia (CNH), while the absence of the CN is referred to as cochlear nerve aplasia (CNA). The cochlear implant (CI) functions by electrically stimulating spiral ganglion neurons (SGNs) and the auditory signals provided by the CI are transmitted to the auditory brainstem via the cochlear branch of the vestibulocochlear nerve. CND was originally considered a contraindication to CI surgery owing to poor outcomes.^3^ Moreover, the possibility of concurrent inner ear malformation (IEM) is much greater in patients with CND than in those without CND,^4^ which further limits CI outcomes. However, patients with CND and severe‐to‐profound hearing loss reportedly show significant auditory improvement with CIs^5^, ^6^ even in cases in which the CN cannot be detected on preoperative imaging. While many patients with CND can benefit from CIs, some patients do not.^6^

The presence of a residual hearing threshold, which represents the integrity of the auditory central conduction pathways, is one of the most important prognostic factors correlated with CI outcomes.^7^ The ability to perceive speech and use language has been a prevalent theme in previous studies of the outcomes of CI in patients with residual hearing, as most of these patients show an improvement in open‐set auditory skills and a significant improvement in language development after CI surgery.^8^ Moreover, improvements in the perception of speech in difficult sound environments have been reported.^9^

However, as a result of limited residual hearing, >70% of patients with CND have hearing level ≥110 dB^4^ and only approximately 50% of patients with CND achieve speech discrimination.^10^ CI outcomes reportedly cannot be explained by the average residual hearing level in patients with CND.^11^, ^12^ The reason for this finding might be that the influence of limited residual hearing on the development of auditory pathways is not significant. Therefore, for groups with minimal residual hearing, indicators other than residual hearing thresholds should be considered to better distinguish hearing differences.

Herein, we hypothesized that both a greater level and a wider frequency range of residual hearing in patients with CND are associated with better CI outcomes. We thus analyzed the correlation between residual hearing and audiologic outcomes in a relatively large study sample.

## 2 MATERIALS AND METHODS

### 2.1 Patients

We retrospectively analyzed the data of 57 patients with CND and profound‐to‐complete SNHL who underwent CI surgery at Tongren Hospital in Beijing between January 2015 and December 2019. All patients underwent unilateral CI surgery. The ear with better residual hearing with hearing aids (HAs) was selected for CI surgery. The use of human subjects in this study was approved by the Research Ethics Board of Tongren Hospital, Beijing, China.

The inclusion criteria were as follows: (1) severe‐to‐complete SNHL, (2) preoperative direct oblique sagittal 3D MRI scans perpendicular to the long axis showing either a thin CN or no CN at the fundus of the internal auditory canal (IAC), (3) no syndromes, (4) a minimum of 3 months of experience wearing HAs and language training before CI surgery (CI was indicated if the patients responded to sound), and (5) successful CI surgery and activation at 1 month postoperation.

### 2.2 Preoperative auditory evaluation

The diagnostic protocol for children with suspected hearing loss involves audiological tests, including measurements of the behavioral observation audiometry (BOA), acoustic emittance, distortion product optoacoustic otoacoustic emission, auditory brainstem response, cochlear microphonics, and 40‐Hz auditory‐evoked related potential. BOA is used to observe changes in auditory behavior in response to stimuli in infants and young children aged <6 months. Visual reinforcement audiometry trains children to establish conditioned reflexes and turn to a light box when hearing the stimulus sound and is suitable for children aged 7 months to 2.5 years. Play audiometry involves simple games during which children respond clearly upon hearing sounds.

Hearing levels were recorded at 0.5, 1, 2, and 4 kHz using BOA. A lack of measurable response was considered indicative of no residual hearing. To calculate the average hearing level, a lack of measurable response was assumed to be 5 dB‐HL greater than the maximum output of the audiometer (125 dB). The average hearing threshold was averaged across 0.5, 1.0, 2.0, and 4.0 kHz on BOA. Hearing impairment was classified in accordance with the World Report on Hearing by the World Health Organization in 2021 as follows: mild (20–35 dB), moderate (35–50 dB), moderately severe (50–65 dB), severe (65–80 dB), profound (80–95 dB), and complete or total hearing loss (≥95 dB).

Due to a high percentage of complete or total hearing loss in our cohort, patients were divided into four groups according to the hearing level as follows: 80–95, 95–110, 110–120, and >120 dB. Based on the measured response at each frequency, patients were divided into three groups according to the residual hearing as follows: entire spectrum hearing, partial spectrum hearing, and no spectrum hearing. Entire spectrum hearing indicated that patients responded at all frequencies during the tests; partial spectrum hearing indicated that patients did not respond to at least one frequency during the tests, and no spectrum hearing indicated that patients did not respond to any of the frequencies during the tests.

### 2.3 Radiographic examinations

High‐resolution computed tomography (HRCT) was performed using a 64‐slice computed tomography (CT) scanner (Philips Brilliance 64, Philips Medical Systems, Best, Netherlands). Volumetric acquisitions were contiguously reconstructed using a 1 mm‐slice thickness throughout the temporal bone. HRCT was used to evaluate IEM according to Sennaroglu's classification.^13^ Moreover, IAC and bony cochlear nerve canal (BCNC) stenosis are suggestive of CND. IAC stenosis generally refers to an IAC diameter <2 mm^14^ and BCNC stenosis generally refers to a BCNC diameter <1.5 mm.^15^ Using CT images, the diameter of the BCNC, the width of the canal at the midportion of the IAC fundus, and the widest diameter of the IAC were measured. MRI was performed using a 1.5‐Tesla scanner (GE Healthcare, Milwaukee, WI, USA) with matched 8‐channel phased array coils. The protocol was designed to obtain not only routine axial and coronal unenhanced T2‐weighted and axial T1‐weighted images but also temporal bone images using axial 3D fast imaging employing steady‐state acquisition. The CN runs along the fundus of the IAC to the base of the modiolus through the CNC. Three‐dimensional (3D) MRI scans were analyzed to determine the CN conditions. Two radiologists separately reviewed all the MRI scans.

### 2.4 Evaluation of CI Outcomes

Auditory performance before and 2 years after CI surgery was evaluated using categorical auditory performance (CAP), speech intelligibility rating (SIR), the Infant–Toddler Meaningful Auditory Integration Scale (IT‐MAIS) for patients aged <3 years, and the meaningful use of speech scale (MUSS).

CAP is an index with eight levels of sound perception (0–7) ranging from “no awareness of the environment” (0) to the “use of telephone with known users” (7). CAP is intended to reflect the real‐life auditory capabilities of children. The SIR is a highly reliable and efficient measure of children's speech production in real‐life situations and ranks a child's spontaneous speech into five categories ranging from “connected speech is unintelligible” (1) to “connected speech is intelligible to all listeners” (5). The IT‐MAIS with scores ranging from 0 to 40 is used to assess auditory performance including changes in vocalization, spontaneous alerting to sounds, and the ability to derive meaning from sounds. The MUSS is a parental reporting scale comprising 10 questions that is used to determine the frequency of use of speech in children's day‐to‐day behavior.

### 2.5 Data and analysis

SPSS statistics software (version 17.0; SPSS Inc., Chicago, IL, USA) was used for data analysis. Descriptive statistics were used to determine the medians, ranges, means, and standard errors of the mean (SEMs). The difference between pre‐ and post‐CI surgery audiologic performance was determined using a paired‐samples *t*‐test. An analysis of variance was performed to determine the differences among the residual hearing groups. All statistical tests were two‐tailed, and *p* < .05 was considered significant.

## 3 RESULTS

### 3.1 Participants

Fifty‐seven patients (33 males and 24 females) with CND who underwent CI surgery were included in the study. All patients had congenital SNHL and had failed newborn hearing screening. Custom electrodes were used for patients with modiolar deficiency‐type IEM (i.e., those with common cavity) and lateral wall electrodes were used for patients with normal cochlea. The mean age (±SEM) at implantation was 32.72 ± 2.17 months (range, 7–59 months). All patients underwent unilateral CI surgery. The ear with better residual hearing with HAs was selected for CI surgery. The left and right ears were implanted in 39 and 18 patients, respectively. Thirty patients were implanted with MED‐EL (Innsbruck, Austria) devices, 16 with Cochlear (Melbourne, Australia) devices, nine with AB (Sonova; Stafa, Switzerland) devices, and two with Nurotron (Hangzhou, China) devices. Sixteen patients (28.1%) had IEM. Fifteen patients (26.3%) had a narrow IAC. Besides the patients with common cavity and incomplete partition‐III (IP‐III), 45 (90.0%, 45/50) of the patients had a narrow BCNC. Twenty‐one (36.8%) patients were fitted with CIs bimodally. The patients' demographic information is shown in Tables 1 and 2.

**TABLE 1:** Demographic characteristics

Pt	Age at CI (mo)	CI side	Cochlea type	CNH/CAN	BCNC	IAC	Contra HA	CI type	Insertion
1	12	L	CH	CNA	S	N	Yes	Med‐El	T
2	7	L	N	CNA	S	N	No	AB	T
3	34	L	N	CNA	S	N	No	Med‐El	T
4	13	L	N	CNA	S	S	No	Cochlear	T
5	25	R	CC	CNA		S	No	Med‐El/CMD	P
6	56	L	CC	CNA		N	Yes	Med‐El/CMD	T
7	29	R	CC	CNA		N	No	Med‐El/CMD	P
8	33	R	N	CNH	S	N	No	AB	T
9	14	R	N	CNA	S	N	Yes	Cochlear	T
10	35	L	N	CNA	S	N	Yes	Cochlear	T
11	14	L	N	CNA	S	N	Yes	AB	T
12	36	L	CC	CNA		N	Yes	Med‐El/CMD	T
13	28	R	CC	CNA		N	Yes	Cochlear	T
14	54	L	N	CNA	S	N	No	Med‐El	T
15	12	R	N	CNH	S	N	No	Cochlear	T
16	38	R	CH	CNA	S	N	Yes	Med‐El	T
17	55	L	N	CNA	S	N	No	AB	T
18	16	L	N	CNA	S	S	No	Cochlear	T
19	59	R	IP‐I	CNA	S	N	No	Med‐El	T
20	45	L	N	CNA	S	S	No	Med‐El	T
21	13	R	N	CNA	S	N	Yes	Cochlear	T
22	17	L	N	CNA	S	S	Yes	Cochlear	T
23	27	L	N	CNA	S	S	No	Med‐El	T
24	40	L	N	CNA	S	N	No	Med‐El	T
25	37	L	N	CNA	S	N	Yes	Cochlear	T
26	8	L	N	CNA	S	S	No	Cochlear	T
27	12	L	IP‐I	CNA	S	N	No	Cochlear	T
28	10	L	IP‐I	CNA	S	N	No	Med‐El	T
29	37	R	N	CNA	S	N	Yes	AB	T
30	36	R	CH	CNA	S	N	No	Med‐El/CMD	P
31	43	L	CH	CNA	S	N	No	Med‐El/CMD	T
32	56	L	IP‐I	CNA	S	N	Yes	Med‐El	T
33	54	R	N	CNA	S	S	Yes	Med‐El	T
34	14	R	N	CNA	S	N	No	Med‐El	T
35	57	L	N	CNA	S	N	No	Med‐El	T
36	27	R	N	CNA	S	S	No	AB	T
37	21	L	CC	CNA		S	No	Med‐El/CMD	T
38	16	L	N	CNA	S	N	No	AB	T
39	52	L	N	CNA	S	N	Yes	AB	T
40	29	L	IP‐II	CNA	S	S	No	Med‐El	T
41	55	R	N	CNA	S	N	No	Cochlear	T
42	25	L	N	CNA	S	S	No	Med‐El	T
43	16	R	N	CNA	S	S	No	Cochlear	T
44	47	L	N	CNA	N	N	Yes	Med‐El	T
45	13	R	N	CNA	N	N	Yes	Med‐El	T
46	57	L	N	CNA	N	N	No	Cochlear	T
47	44	L	N	CNA	N	N	No	AB	T
48	32	L	N	CNA	S	S	Yes	Med‐El	T
49	47	L	N	CNA	S	S	No	Med‐El	T
50	26	L	N	CNA	S	N	Yes	Cochlear	T
51	14	R	N	CNA	S	N	Yes	Cochlear	T
52	36	L	N	CNA	S	N	Yes	Med‐El	T
53	56	L	N	CNH	S	N	No	Med‐El	T
54	56	L	N	CNA	S	N	No	Nurotron	T
55	27	L	N	CNA	N	N	No	Med‐El	T
56	57	L	IP‐III	CNA		N	No	Med‐El	T
57	36	L	N	CNA	S	N	No	Nurotron	T

Abbreviations: CC, common cavity; CH, cochlear hypoplasia; CMD, custom‐designed electrode; CNA, cochlear nerve aplasia; CNH, cochlear nerve hypoplasia; IP, incomplete partition; L, left; N, normal; P, partial; R, right; S, stenosis; T, total.

**TABLE 2:** Residual hearing and CI outcomes

Pt	Hearing level	Hearing range	CAP		SIR		IT‐MAIS		MUSS	
			0 m	24 m	0 m	24 m	0 m	24 m	0 m	24 m
1	118.75	P	0	2	1	2	2	25	0	11
2	125	N	0	5	1	1	0	28	0	5
3	87.5	E	0	5	1	3	3	39	0	21
4	125	N	0	2	1	1	0	20	0	2
5	111.25	E	1	7	1	2	3	32	1	6
6	125	N	0	5	1	2	0	33	0	15
7	125	N	0	4	1	2	0	31	0	9
8	115	E	0	5	1	2	0	40	0	6
9	98.75	E	0	3	1	4	3	35	1	23
10	82.5	E	0	7	1	1	6	20	1	2
11	125	N	0	2	1	1	0	4	0	6
12	125	N	1	5	1	2	0	30	0	17
13	93.75	E	1	6	1	1	0	28	0	6
14	116.25	P	0	3	1	3	0	34	0	26
15	83.75	E	1	5	1	4	0	33	1	32
16	125	N	0	3	1	1	0	11	0	2
17	125	N	1	5	1	2	6	12	6	1
18	125	N	0	2	1	1	0	5	1	1
19	125	N	0	5	1	2	0	34	0	12
20	80	E	1	5	1	2	3	30	4	10
21	97.5	E	2	5	1	3	5	32	2	24
22	118.75	P	0	3	1	2	0	6	0	2
23	100	E	1	5	1	3	6	39	0	33
24	95	E	1	5	1	2	10	38	4	10
25	121.25	P	0	3	1	2	0	36	0	28
26	125	N	0	4	1	2	1	27	1	4
27	110	E	1	6	1	3	0	36	0	22
28	86.25	E	0	4	1	2	7	39	2	10
29	101.25	E	0	4	1	2	1	21	0	4
30	125	N	0	3	1	2	0	27	0	11
31	81.25	E	1	4	1	2	8	26	0	14
32	113.75	E	0	5	1	3	0	36	0	24
33	92.5	E	2	5	1	4	0	35	0	18
34	111.25	P	0	3	1	1	0	26	0	5
35	111.25	P	0	4	1	1	0	19	0	8
36	125	N	1	3	1	1	0	23	0	3
37	125	N	1	5	1	2	2	28	3	16
38	123.75	P	1	4	1	1	0	27	0	14
39	112.5	E	1	5	1	2	0	40	0	30
40	106.25	P	2	5	1	2	0	6	0	5
41	98.75	E	0	5	1	1	0	18	0	4
42	116.25	P	0	3	1	2	6	40	5	25
43	87.5	E	1	5	1	2	0	32	0	15
44	125	N	0	2	1	2	0	24	0	4
45	103.75	E	0	5	1	4	6	40	0	33
46	121.25	P	0	3	1	1	0	22	0	18
47	116.25	E	2	5	1	2	6	27	2	14
48	100	E	1	4	1	2	0	21	0	4
49	117.5	P	1	5	1	1	0	4	0	6
50	125	N	1	5	1	3	2	33	0	27
51	125	N	0	2	1	1	0	17	0	3
52	102.5	E	1	6	1	2	2	32	0	30
53	108.75	E	1	5	1	2	0	29	0	15
54	102.5	E	0	3	1	1	0	16	0	3
55	92.5	E	2	4	1	3	0	36	0	18
56	121.25	P	1	5	1	1	0	16	0	3
57	96.25	E	0	5	1	2	5	18	2	8

Abbreviations: CAP, categorical auditory performance; CI, cochlear implant; E, entire spectrum hearing group; IT‐MAIS, infant–toddler meaningful auditory integration scale; MUSS, meaningful use of speech scale; N, no spectrum hearing group; P, partial spectrum hearing group; SIR, speech intelligibility rating.

### 3.2 Audiologic characteristics of patients with CND who underwent CI surgery

**FIGURE 1:**
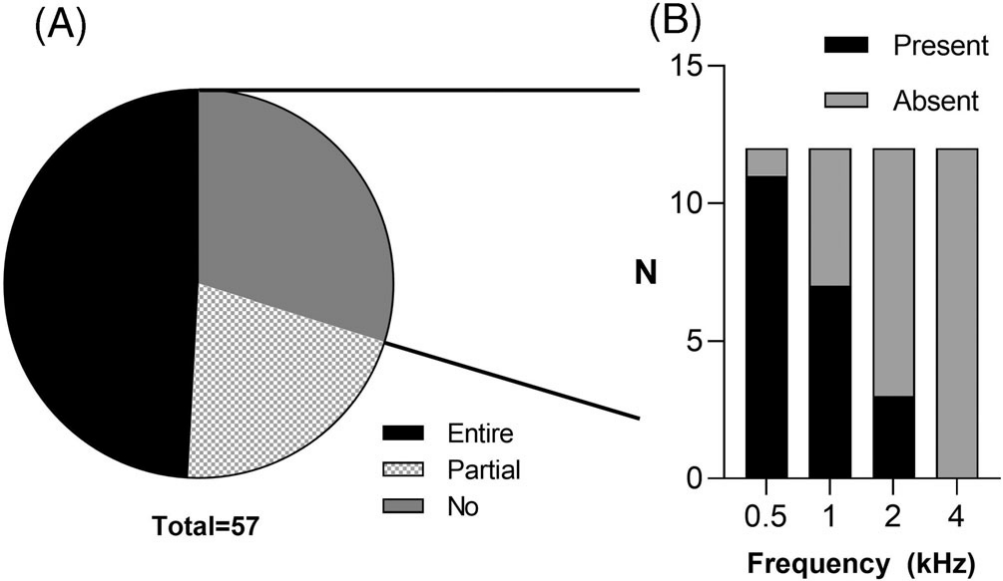
Profile of residual hearing level. A, Scatter plot of the average hearing threshold. B, Number of patients in the 80–95, 95–110, 110–120, and >120 dB groups.

The median average hearing threshold of the patients was 111.25 dB (range, 76.25–125 dB) as shown in Figure 1A. Ten (17.5%) patients had profound (80–95 dB) hearing loss and 47 (82.5%) patients had complete or total hearing loss (≥95 dB). Among the 47 patients with complete or total hearing loss, 13 had a hearing level of 95–110 dB, 17 had a hearing level of 110–120 dB, and 17 had no response to sound without HAs (hearing level > 120 dB) (Figure 1B).

**FIGURE 2:**
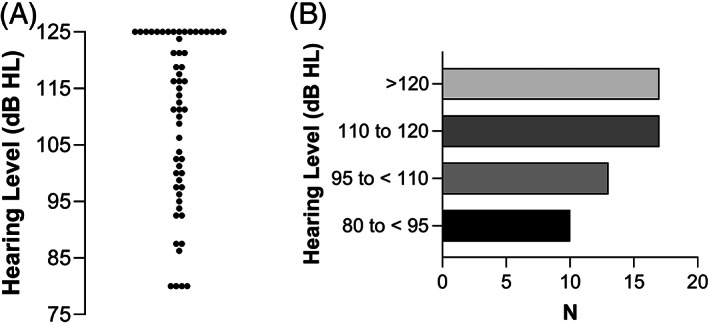
Hearing level at 0.5, 1.0, 2.0, and 4.0 kHz. A, Scatter plot of the hearing threshold. B, Number of patients in the 80–95, 95–110, 110–120 dB, and > 120 dB groups.

The hearing levels at frequencies of 0.5, 1, 2, and 4 kHz are shown in Figure 2 (A and B). Fourteen (24.6%), 10 (17.5%), seven (12.3%), and nine (15.8%) of the 57 patients had a hearing level < 95 dB at frequencies of 0.5, 1, 2, and 4 kHz, respectively. Furthermore, 39 (68.4%), 35 (61.4%), 31 (54.4%), and 28 (49.1%) of the 57 patients had residual hearing at 0.5, 1, 2, and 4 kHz, respectively.

**FIGURE 3:**
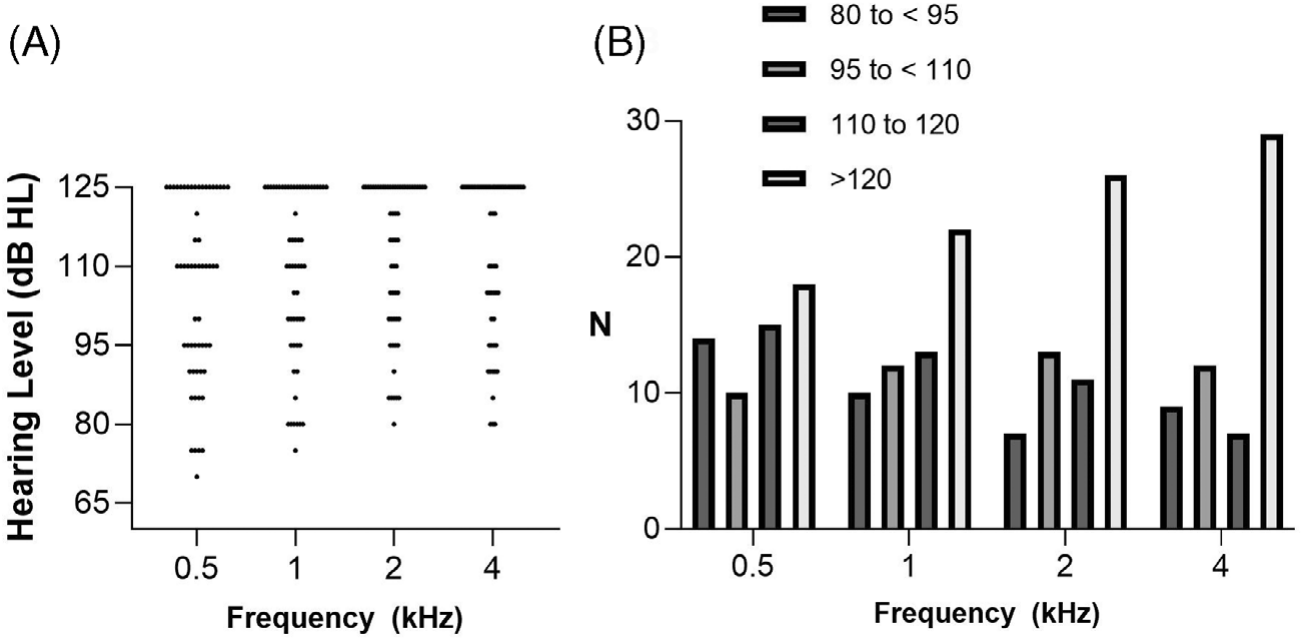
Profile of the residual hearing spectrum. A, Composition of the hearing spectrum subgroups. Entire spectrum hearing involves responses at all frequencies during the tests, partial spectrum hearing involves response at a minimum of one frequency, and no spectrum hearing involves no response at any of the frequencies during the tests. B, Summary data of responses at each frequency in the partial spectrum hearing group.

Among the 57 patients, 28 (49.1%), 12 (21.1%), and 17 (29.8%) were included in the entire spectrum, partial spectrum, and no spectrum hearing groups, respectively (Figure 3A). In the partial spectrum hearing group (Figure 3B), 12 (100%) patients showed no residual hearing at 4 kHz, nine (75%) displayed no residual hearing at 2 kHz, five (41.7%) displayed no residual hearing at 1 kHz, and one (8.3%) displayed no residual hearing at 0.5 kHz.

### 3.3 Correlation between residual hearing and auditory performance after CI surgery

**FIGURE 4:**
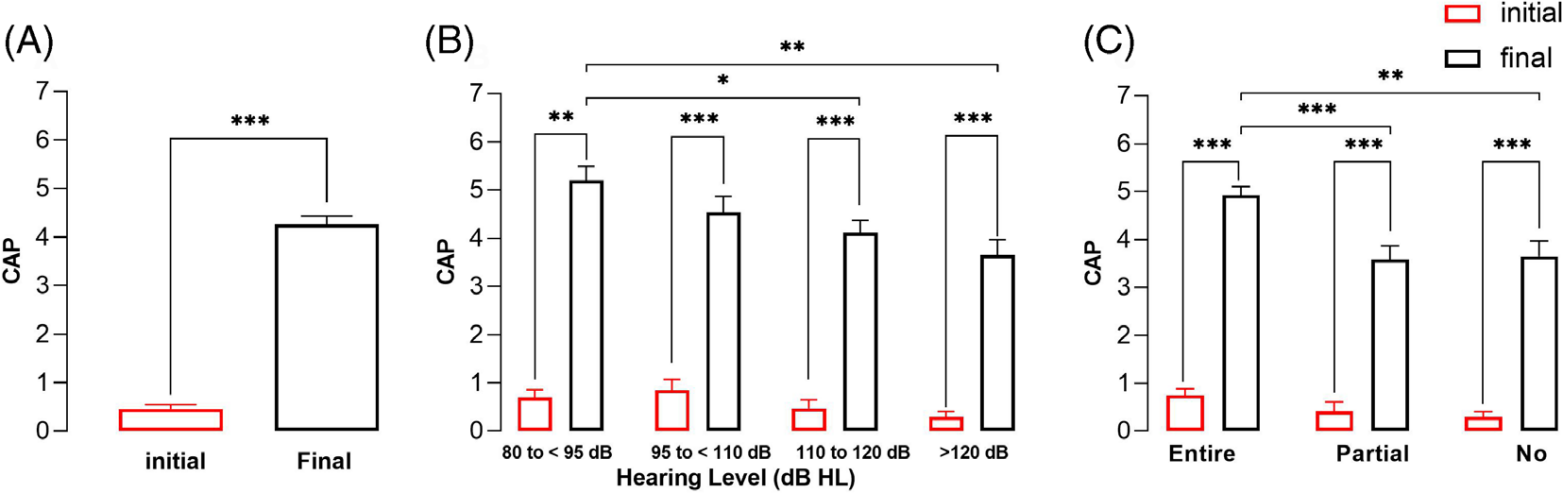
CAP scores. A, overall CAP scores. B, CAP scores of the residual hearing level subgroups. C, CAP scores of the residual hearing spectrum subgroups. Error bars are for SEMs. Statistical significance is indicated by * (*p < .05; **p < .01; ***p < .001).

The CAP and IT‐MAIS were used to assess auditory performance before and after CI surgery. Overall, the CAP score before and after CI surgery was 0.54 ± 0.09 and 4.26 ± 1.25, respectively. The difference was significant, as shown in Figure 4A (*p* < .001). All hearing level subgroups showed significant improvements (*p* < .05), as shown in Figure 4B. Two years after CI surgery, the CAP score was significantly higher for 80–95 dB than 110–120 and >120 dB (*p* < .01 and *p* < .05, respectively). All hearing spectrum subgroups (Figure 4C) showed significant improvements in the CAP score (*p* < .001). Compared to the entire spectrum hearing group, the partial spectrum hearing and no spectrum hearing groups showed significantly poorer postoperative CAP scores (*p* < .001 and *p* < .01, respectively).

**FIGURE 5:**
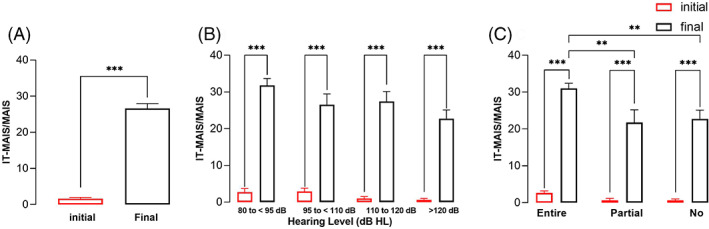
IT‐MAIS/MAIS scores. A, overall IT‐MAIS/MAIS scores. B, IT‐MAIS/MAIS scores of the residual hearing level subgroups. C, IT‐MAIS/MAIS scores of the residual hearing spectrum subgroups. Error bars are for SEMs. Statistical significance is indicated by * (**p < .01; ***p < .001).

All patients showed significant improvements in IT‐MAIS scores (*p* < .001) from 1.63 ± 0.35 to 26.60 ± 1.34 (Figure 5A). All hearing level and hearing spectrum subgroups except for the hearing level group showed statistically significant differences between preoperative and postoperative IT‐MAIS scores (*p* < .001 for all) (Figure 5B,C). The entire spectrum hearing group had significantly higher scores than the partial spectrum hearing and no spectrum hearing groups (*p* < .01).

### 3.4 Correlation between residual hearing and speech perception after CI surgery

**FIGURE 6:**
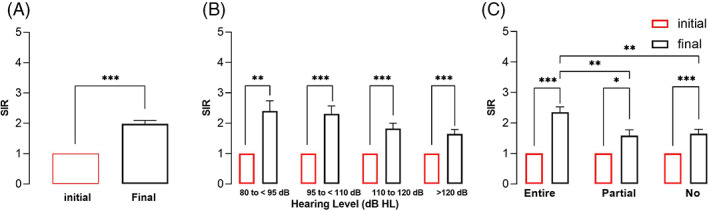
SIR scores. A, overall SIR scores. B, SIR scores of the residual hearing level subgroups. C, SIR scores of residual hearing spectrum subgroups. Error bars are for SEMs. Statistical significance is indicated by * (**p < .01; ***p < .001).

We used the SIR and MUSS scores to evaluate speech performance. All patients had a preoperative SIR score of 1. The postoperative SIR score was 1.98 ± 0.11. The SIR scores for the overall cohort and hearing level and hearing spectrum subgroups were significantly different before and after CI surgery (*p* < .01) (Figure 6A–C). However, the SIR scores were not significantly different among the hearing level subgroups. In contrast, among the hearing spectrum subgroups, the entire spectrum hearing group had significantly higher SIR scores than the partial spectrum hearing and no spectrum hearing groups (*F* = 6.301, *p* < .01).

**FIGURE 7:**
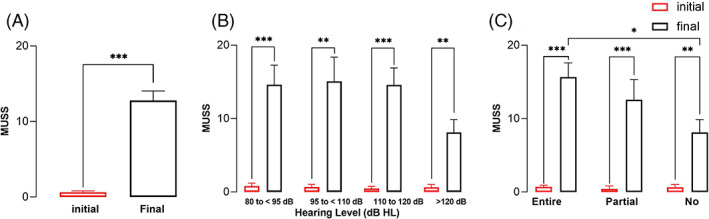
MUSS scores. A, overall MUSS scores. B, MUSS scores of the residual hearing level subgroups. C, MUSS scores of the residual hearing spectrum subgroups. Error bars are for SEMs. Statistical significance is indicated by * (*p < .05; **p < .01; ***p < .001).

The MUSS scores showed significant improvements over time for the overall cohort from 0.63 ± 0.18 to 12.77 ± 1.28 (*p* < .01) (Figure 7A). A weak correlation among the hearing level subgroups was observed for the MUSS score (Figure 7B). Among the hearing spectrum subgroups, the no spectrum hearing group had significantly poorer MUSS scores than the entire spectrum hearing group (Figure 7C).

## 4 DISCUSSION

The present study demonstrated that the condition of residual hearing was significantly associated with postoperative audiologic outcomes after CI surgery.

Patients with CND display limited residual hearing. In a previous study, >70% of ears with CND showed profound hearing loss.^4^ The presence of residual hearing is an indication of the integrity of the neural pathways including SGNs and the CNs. In patients with CND, the CN is thin or absent, limiting the transmission of electrical signals to the auditory brainstem. Furthermore, approximately 30% of patients with CND present with IEM,^4^ which is associated with a significantly reduced number of SGNs. In the current cohort, 28.1% of the patients had IEM.

Due to the limitations of imaging techniques that are currently available, the CN may not be directly visible on MRI. The IAC and BCNC indirectly determine the condition of the CN. IAC stenosis is often considered to be associated with CND.^16^, ^17^ However, a growing number of studies have shown that a narrow IAC is not always indicative of CND.^6^, ^18^ BCNC stenosis is a more sensitive indicator of CND than IAC stenosis. Chung et al.^12^ found that the incidence of CND was much higher in patients with BCNC stenosis (76%) than in patients with a normal BCNC (21%) and the width of the BCNC in patients with CND (1.11 mm) was significantly smaller than that in patients with normal CNs (2.08 mm). In a previous study,^19^ the BCNC width was used to correctly identify CND with 84% sensitivity and 98% specificity, while the IAC width showed a sensitivity of 44% and a specificity of 98%. In our study, approximately 30% of the patients had a narrow IAC and most of the patients had a narrow BCNC.

Children with residual hearing have a higher likelihood of early auditory development than those with no measurable thresholds, leading to better auditory performance after CI surgery.^7^ Although some patients had no residual hearing before the surgery, all responded to sound after HA training indicating that the SGNs and central auditory conduction pathways are present. All patients showed significant improvements in auditory and speech perception. In previous studies, CI outcomes could not be explained by residual hearing levels due to the limited hearing levels and delayed audiologic progression associated with patients with CND.^11^, ^12^ To discriminate CI outcomes in patients with complete or total hearing loss, we divided patients with CND into four groups according to hearing level. The present study showed that only postoperative CAP scores had significantly positive correlations with hearing level; patients with a hearing level <95 dB showed higher CAP scores than those with a hearing level >110 dB. However, no significant differences were observed in the postoperative IT‐MAIS, SIR, and MUSS scores. Hearing levels were positively correlated with postoperative audiology performance, but weakly correlated with postoperative speech perception.

The range of residual hearing could be used to better predict postoperative CI outcomes. A previous study demonstrated differences in the performance of children with 70 dB‐HL of residual hearing (up to 1 kHz) and those with only low‐frequency residual hearing (up to 0.5 kHz) and reported that children with a wider frequency range of hearing showed better performance.^20^ The present study showed that patients with entire spectrum hearing had significantly better CAP, SIR, and IT‐MAIS scores than those with partial spectrum hearing and significantly better CAP, SIR, IT‐MAIS, and MUSS scores than patients without spectrum hearing. The range of residual hearing was positively correlated with both postoperative audiologic performance and speech perception.

In cases where the assessment process reveals no evidence that a CI would provide any benefit or would provide inadequate benefits, an auditory brainstem implant (ABI) should be considered. CND is considered an indication for ABI surgery.^13^ However, ABIs are neurosurgical procedures associated with a risk of serious complications such as cerebrospinal fluid leaks, meningitis, intracranial bleeding, strokes, cranial nerve damage, and even death.^21^ CIs should be the first approach for patients with CND who respond to sound after an HA trial and language training. Herein, all patients demonstrated improvement in auditory performance and speech perception after CI surgery. However, we recommend that clinicians pay close attention to the progress of patients with CND without spectrum hearing. If patients have limited auditory and speech progress at 6 months to 1 year after CI surgery, a contralateral ABI should be considered.

## 5 CONCLUSION

This is the first study to assess the correlation between residual hearing and auditory performance after CI surgery in patients with CND. In addition to the average hearing threshold, close attention should be paid to the range of residual hearing. Residual hearing, especially high‐frequency residual hearing, is poor in patients with CND, and the CI outcomes in patients with CND are variable. The current study showed that postoperative audiologic outcomes in patients with CND were significantly associated with the range of residual hearing. Furthermore, the evaluation of residual hearing using behavioral audiometry may provide useful information that can facilitate preoperative counseling for these patients.

## AUTHOR CONTRIBUTIONS

Simeng Lu analyzed the patients' cochlear nerve deficiency data and was a major contributor to the writing of the manuscript. Xingmei Wei substantively revised the manuscript. All authors read and approved the final manuscript.

## CONFLICT OF INTEREST

None of the authors received any other funding and none have financial relationships or conflicts of interest to disclose.

## ETHICS STATEMENT

This study was approved by the Research Ethics Board of Tongren Hospital, Beijing, China.

## CONSENT STATEMENT

The parents of the patients were fully informed about the evaluation prior to participation. All the parents provided written informed consent and participated in the evaluation free from coercion. The patients' information was protected. Written informed consent for the publication of details regarding the patients' clinical status was obtained from the parents.

## Data Availability

The datasets used and analyzed during the current study are available from the corresponding author upon reasonable request.
